# Phytotoxicity and metal mobility in soils contaminated with mine tailings

**DOI:** 10.1007/s10653-026-03203-x

**Published:** 2026-04-24

**Authors:** Andressa Cristhy Buch, Maria Elizabeth Fernandes Correia, Júlia Carina Niemeyer, Douglas Brian Sims, Eduardo Duarte Marques, Krishna Kumar Yadav, Ahmad J. Obaidullah, Camila Rodrigues e Silva, Emmanoel Vieira da Silva-Filho

**Affiliations:** 1 Department of Environmental Geochemistry, Universidade Federal Fluminense, Outeiro São João Baptista, S/N, Centro, 24020-007 Niterói, Rio de Janeiro Brazil; 2 Brazilian Agricultural Research Company (EMBRAPA), 23897-000 Seropédica, Rio de Janeiro Brazil; 3 Postgraduate Program in Agricultural and Natural Ecosystems, Universidade de Santa Catarina, Campus de Curitibanos, Rod. Ulysses Gabordi, km 3, 89520-000 Curitibanos, Santa Catarina Brazil; 4 Department of Physical Sciences, College of Southern Nevada, North Las Vegas, Nevada 89030 USA; 5 Service Geological Survey of Brazil/Company of Research of Mineral Resources (SGB/CPRM), Av. Brasil, 1731, Funcionários, Belo Horizonte, Minas Gerais 30140-002 Brazil; 6 Department of VLSI Microelectronics, Saveetha School of Engineering, Saveetha Institute of Medical and Technical Sciences (SIMATS), Saveetha University, Chennai, Tamil Nadu 602105 India; 7 Environmental and Atmospheric Sciences Research Group, Scientific Research Center, Al-Ayen University, Nasiriyah, Thi-Qar Iraq; 8 Department of Pharmaceutical Chemistry, College of Pharmacy, King Saud University, P.O. Box 2457, 11451 Riyadh, Saudi Arabia; 9 Department of Chemistry, Graphic Era University, Dehradun, 248002 Uttarakhand India

**Keywords:** Chlorophyll inhibition, Dam collapse, Germination, Soil–plant interface, Stomata movements

## Abstract

The consumption of vegetables is essential for human health. However, in regions affected by mining activities, this practice may pose serious risks due to potential heavy metal contamination at the soil–plant interface. This study evaluated the phytotoxicity and metal mobility in lettuce cultivated in mine tailing-contaminated soils after the Córrego do Feijão dam failure (Brumadinho, Minas Gerais, Brazil). The increase in soil metal concentrations negatively influenced morphological parameters such as root and shoot length, fresh biomass, germination, chlorophyll content, and the pattern and frequency of stomata on the leaf surface. Translocation factors of Fe, Ni, Mn, Cd, Cu, Pb, and Zn from roots to shoots of lettuce indicated significant risks to human health. In shoots, the concentrations of As, Cd, Pb, and Cu exceeded the maximum permissible limits for human consumption in lettuce and other leafy Asteraceae by up to 407-, 98-, 5,483-, and 28-fold, respectively. These results highlight the urgent need for mitigation, remediation, and protective strategies to safeguard human and animal health in mining regions, particularly where mining, agriculture, and livestock production coexist.

**Supplementary Information:**

The online version contains supplementary material available at 10.1007/s10653-026-03203-x.

## Introduction

The mining sector has expanded markedly in recent decades, driven by increasing global demand for minerals, particularly from developing economies and the energy transition. While this growth generates substantial economic benefits in resource-rich regions, it also poses environmental and social challenges, including deforestation, community displacement, and potential risks to human health, underscoring the need for robust governance and sustainable practices (Worlanyo & Jiangfeng, 2021).

Metal contamination in mining regions can persist for centuries, degrading water, soil, and air quality and causing long-term ecological and health impacts (Islam & Murakami, 2021). Failures of mine tailings dams often exacerbate these effects, leading to extensive soil contamination by toxic elements (Armstrong et al., 2019; Kossoff et al., 2014). Soil serves as a primary sink for contaminants, where metals may be retained, transformed, or mobilized to other environmental compartments, thereby affecting diverse biological receptors (Buch et al., 2021; Kabata-Pendias, 2011). Accordingly, environmental risk assessments of metal-contaminated soils should integrate ecological and phytotoxicity endpoints, with bioassays serving as key tools to support regulatory frameworks (FCSAP, 2010; Bagur-González et al., 2011; ISO, 2022; Buch et al., 2024a, 2024b).

Plants readily accumulate metals, particularly in naturally enriched or anthropogenically contaminated soils, such as those affected by mine tailings (Cobb et al., 2000; Islam et al., 2020; Parviainen et al., 2022). Consequently, agricultural production in mining-impacted areas may facilitate metal transfer through the food chain, posing ecological and potential human health risks (Buch et al., 2024a, 2024b; Dutta et al., 2021). Metal uptake, translocation, and distribution in plants depend on species traits, metal speciation, and environmental conditions, including soil and climate (Buch et al., 2018, 2020; Coelho et al., 2020). Although vegetables assimilate essential nutrients, they may also accumulate toxic elements such as arsenic, lead, and cadmium through physiological and metabolic pathways that can be disrupted by contamination (Antoniadis et al., 2016; Rehman et al., 2021).

Leaf pigments, particularly chlorophyll *a* and *b*, are sensitive indicators of soil quality and metal uptake at the soil–plant interface. Located in chloroplast thylakoid membranes, these pigments drive photosynthesis by converting light energy into chemical energy (Gross, 1991). Excessive concentrations of essential and non-essential metals induce ionic stress and disrupt multiple physiological processes, including stomatal regulation, which is governed by guard cell turgor and influenced by water status, metal toxicity, CO₂ concentration, and light (Raschke, 1975; Rucińska-Sobkowiak, 2016).

In Brazil, mining is a major economic activity, particularly in Minas Gerais State. The Iron Quadrangle is a globally significant polymineral province with large reserves of iron ore, manganese, nickel, aluminum, and gold (CPRM, 2019a). On January 25, 2019, the iron ore tailings dam at the Córrego do Feijão Mine in Brumadinho (Minas Gerais, Brazil), operated by Vale S.A., collapsed, releasing approximately 12 million m^3^ of tailings. The resulting slurry traveled more than 300 km, directly affecting 18 municipalities, causing 278 fatalities, and severely damaging aquatic and terrestrial ecosystems (CPRM, 2019a, 2019b). An estimated 1.8 million hectares were impacted, including 300 ha of native vegetation, with substantial losses to agricultural production (CPRM, 2020; Diário do Comércio, 2019).

Metals released from mine tailings act as persistent chemical stressors that degrade ecological quality, impair plant growth, and threaten primary and secondary consumers, with implications for human exposure. In this context, the present study evaluates the bioaccumulation, translocation, and phytotoxic effects of metals in lettuce cultivated in soils affected by one of the most severe mining-related environmental incidents recorded worldwide.

## Material and methods

### Study areas/sample collection of test soil

**Fig. 1:**
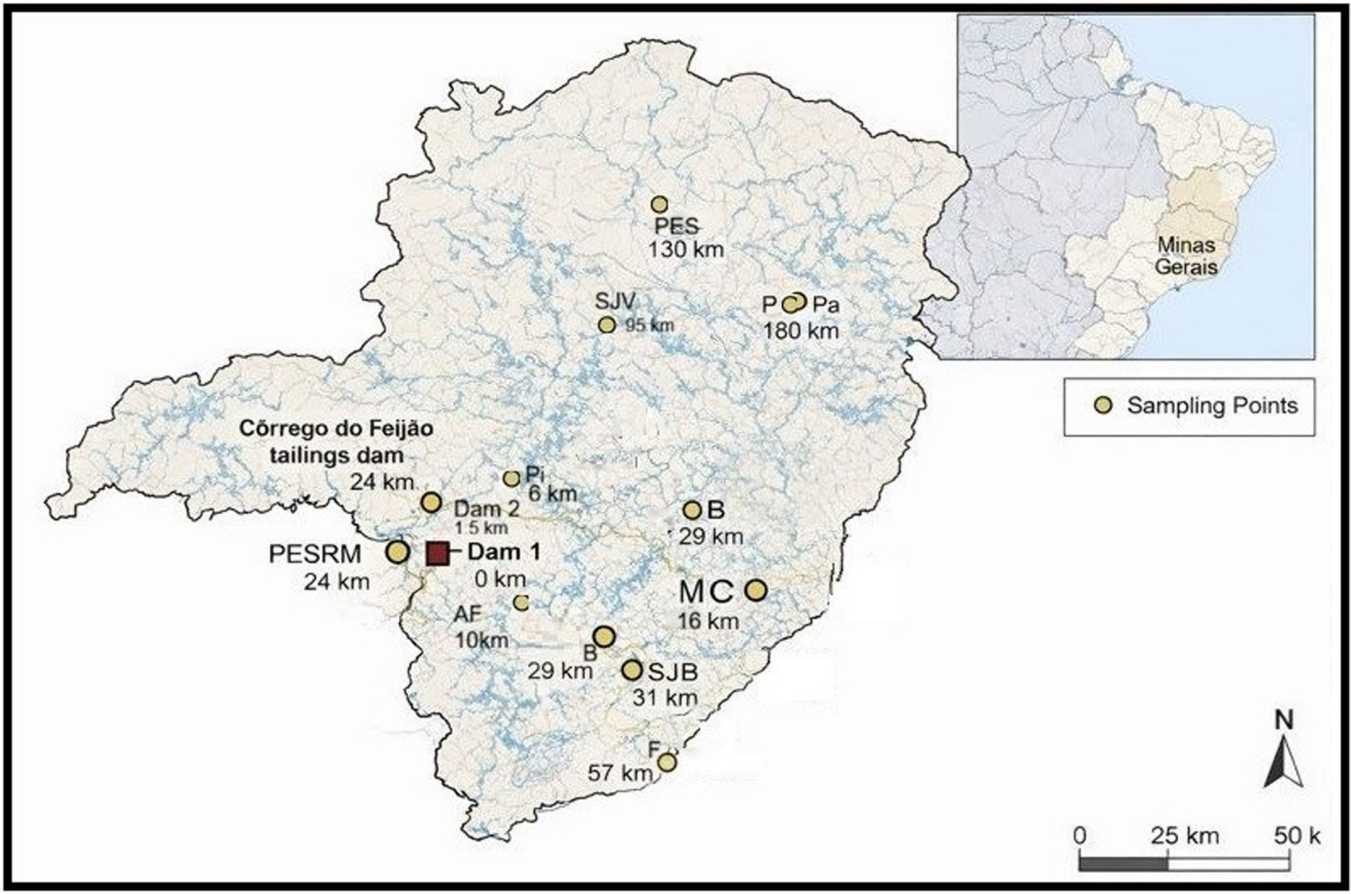
Spatial distribution of sampling points and their distances from the Córrego do Feijão Mine tailings dam (Minas Gerais, Brazil)

Eleven test soils from riparian areas affected by CFM tailings were used for lettuce cultivation: Dam 1 and Dam 2 (where the CFM dam is located in the Municipality of Brumadinho, MG), Pinheiros (Pi), Alberto Flores (AF), Mário Campos (MC), Betim (B), São Joaquim de Bicas (SJB), Florestal (F), São José da Varginha (SJV), Paraopeba (Pa), Pompéu (P). Control soils from two reference areas (non-impacted sites by mining activities) were also used: (i) Parque Estadual da Serra do Rola-Moça (PESRM) located in the Nova Lima municipality in MG State (24 km from the dam rupture) and (ii) Parque Estadual do Sumidouro (PES) situated in an Environmental Conservation Unit in Pedro Leopoldo and Lagoa Santa municipalities in MG State (130 km from the dam rupture). Geographical coordinates and distances of these areas to CFM dam areas can be seen in Fig. 1 and Table S1. Ten subsamples (0–20 cm depth) were collected using a flat hand shovel from a representative grid (20 × 20 m) to compose a composite sample for each area. Sampling was conducted in February 2019, 17 days after the dam collapse, and again in February 2022 at the same previously sampled sites. Soils collected in 2019 and 2022 were used in separate experiments with lettuce plants.

Bioavailability and total concentrations of metals (Al, As, Cd, Cu, Cr, Fe, Pb, Mn, Ni, Ti and Zn) in soil and plant samples were determined after acid digestion in accordance with USEPA Method 3051 (2007). Specifically, 0.5 g of each sample was placed in a Teflon vessel, 10 mL of HNO_3_ was added, and the vessels were sealed and subjected to microwave digestion for 10 min. After cooling, the digested solutions were filtered using Whatman 42 filter paper and brought to a final volume of 50 mL with deionized water in a volumetric flask. Simple extraction was performed by treating the samples with the Mehlich-1 extracting solution (0.05 mol L⁻^1^ HCl + 0.0125 mol L⁻^1^ H₂SO₄; 1:10, w/v) to solubilize the readily available metal fraction. The resulting extracts were filtered and subsequently analyzed by Inductively Coupled Plasma-Mass Spectrometry (ICP-MS) and mercury (Hg) was determined by atomic absorption spectrometry using cold vapor atomization, following USEPA guidelines (1994; 2007). Quality control was based on the certified reference material SRM 2709 (San Joaquin Soil), provided by the National Institute of Standards and Technology (NIST, U.S. Department of Commerce). The average recovery rates for metals ranged from 86 to 97%. The chemical characterization of the test soils is presented in Table 1. Soil pH values were evaluated according to EMBRAPA (2011) and measured potentiometrically using a digital benchtop pH meter (model pH 300 M).

**Table 1:** Total metal concentrations in soils affected by the Córrego do Feijão Mine dam rupture. Al and Fe are expressed as %, while all other metals are reported in mg kg⁻^1^ (dry weight)

	Al		As		Cd		Cr		Cu		Fe		Hg		Mn		Ni		Pb		Ti		Zn	
	2019	2022	2019	2022	2019	2022	2019	2022	2019	2022	2019	2022	2019	2022	2019	2022	2019	2022	2019	2022	2019	2022	2019	2022
	%		mg/kg		mg/kg		mg/kg		mg/kg		%		mg/kg		mg/kg		mg/kg		mg/kg		mg/kg		mg/kg	
PESRM	1.2	1.01	2.01	2.14	0.04	0.03	12.71	11.25	10.14	11.5	15.45	16.1	0.02	0.01	0.43	0.45	4.01	4.12	3.45	3.32	0.01	0.01	28.09	26.98
PES	1.17	1.11	1.98	2.07	0.02	0.04	13.32	8.14	12.02	10.01	16.79	16.7	0.01	0.02	0.55	0.51	3.75	3.52	2.39	2.56	0.01	0.01	30.51	32.10
Dam 1	1.47	1.43	22.32	22.04	1.01	0.92	79.82	79.12	88.75	87.08	28.67	28.31	0.23	0.21	1.63	1.56	34.82	34.08	79.85	74.36	0.05	0.05	79.59	75.11
Dam 2	1.69	1.55	29.21	21.18	0.97	0.84	76.96	75.03	92.43	85,00	28.12	27.21	0.24	0.24	1.74	1.45	34.79	33.19	72.14	73.05	0.05	0.04	85.89	82.65
Pi	1.51	1.47	27.17	20.13	0.79	0.78	71.21	64.12	84.22	85.17	25.69	25.31	0.21	0.21	1.61	1.63	32.05	31.12	76.83	68.24	0.04	0.04	78.31	78.22
AF	1.37	1.22	25.71	29.13	0.75	0.74	69.43	63.17	78.18	73.65	24.91	24.83	0.17	0.19	1.53	1.58	29.63	22.07	59.95	59.56	0.03	0.04	66.11	69.25
MC	1.42	1.36	17.28	18.41	0.69	0.72	65.11	68.45	69.22	77.61	23.47	23.21	0.18	0.18	1.61	1.63	21.22	21.25	40.72	47.18	0.03	0.03	58.13	66.18
B	1.37	1.28	19.93	20.32	0.52	0.71	75.11	78.18	81.02	82.95	22.89	23.05	0.13	0.18	1.56	1.67	30.12	29.67	49.11	51.36	0.03	0.05	80.12	68.93
SJB	1.17	1.11	16.29	17.32	0.51	0.63	47.91	49.12	72.87	71.19	22.95	22.61	0.15	0.16	1.76	1.68	21.7	22.03	29.64	37.18	0.03	0.03	53.8	55.78
F	0.99	1.03	12.52	12.79	0.58	0.48	38.06	38.78	62.12	66.26	18.94	18.71	0.11	0.12	1.46	1.55	19.61	18.25	19.93	20.12	0.03	0.03	62.11	60.17
SJV	1.14	1.08	14.39	14.33	0.48	0.51	42.56	44.17	68.61	61.09	19.65	19.21	0.11	0.13	1.57	1.54	18.71	17.81	19.67	20.53	0.03	0.03	55.82	59.21
Pa	1.06	1.01	14.83	15.07	0.47	0.62	49.53	48.91	77.73	72.12	19.32	18.75	0.18	0.16	1.62	1.66	19.71	18.39	19.56	21.03	0.02	0.03	52.13	53.02
P	0.97	0.94	13.99	14.12	0.46	0.53	39.77	43.18	66.95	66.12	17.81	17.41	0.15	0.14	1.49	1.44	18.61	19.22	19.74	20.12	0.02	0.02	51.53	49.17

Acronyms refer to: PESRM-Parque Estadual da Serra do Rola-Moça; PES-Parque Estadual do Sumidouro; Dam 1 and 2 to dam areas; Pi-Pinheiros; AF-Alberto Flores; MC-Mário Campos; B-Betim; SJB-São Joaquim de Bicas; F-Florestal; SJV-São José da Varginha; Pa-Paraopeba; P-Pompeu

### Soil characterization

A brief soil physical–chemical characterization is shown in Table S2. In general, the soil textures affected by CFM tailings varied from sandy loam to silt loam. In the reference areas, sandy clay texture prevailed. All areas showed acidic pH, with the lowest values noted in areas closest to the broken dam (2.9–4.7). This was also observed for the highest bulk densities up to 4.85 g cm^−3^ and low content of organic matter up to 7.6%. In general, the order of metal concentrations (from high to low) found in the areas was Fe > Mn > Al > Ti > Cu > Zn > Cr > Pb > Ni > As > Sr > Cd > Hg, as shown in Table 1. The higher values were found in areas of Dam 1 and 2; Pi, AF, MC, B, and SJB, whose municipalities are located closer to the broken dam areas. Although in 2022 soil samples, some areas showed slightly higher metal concentrations, for most areas, there were no significant statistical differences between the concentrations for the two sampling years (2019 and 2022). Further details can be found in Buch et al., (2023, 2024a).

### Test species

Lettuce (*Lactuca sativa* Linn.; Asteraceae family) is one of the most consumed vegetables in Brazil and in the world. Crispa variety seeds with a germination rate of 75% and 98% purity were used. Due to its accumulating characteristics, it is known as a bioindicator species of metals in soils, showing high relevance to phytotoxic investigations (Česynaitė et al., 2023). Physiological and morphological changes in lettuce may reflect the environmental changes and bioavailability of metals under various soil conditions (Moreira et al., 2020).

### Experimental design

For the assessment of bioaccumulation, translocation, and phytotoxicity, lettuce plants were grown in glass vase pots (13 cm diameter, and 350 mL capacity) filled with 250 g of test soil. Each pot was considered an experimental unit. Four replicate pots were used for each treatment (test soils from eleven contaminated and two control soils) in each sampling years (2019 and 2022). All parameters were assessed on a per-plant basis across replicates. Bioassays were conducted in a climate chamber at 70 ± 3.2% relative humidity, 20 ± 2 °C temperature, and a 16-h photoperiod with 350 ± 50 µE/m^2^/s.µmol.m^−2^s^−1^ irradiance provided by fluorescent lamps (TL-D 36 W, Philips). All measurements and observations were compared with those of uncontaminated control plants (from the PESRM and PES areas).

### Bioaccumulation and translocation factors

Roots and shoots were separated and lyophilized for the determination of metals by digestion in accordance with Method 3051 (USEPA, 2007). Afterward, they were analysed by inductively coupled plasma mass spectrometry (ICP-MS) according to USEPA (1994 and 2000) and by atomic absorption spectrometry using cold vapor atomization for Hg according to USEPA (1986). The bioaccumulation and translocation factors were measured after 30 days of exposure in the germination bioassay (OECD 208, 2006).

The bioaccumulation factor (BAF) and translocation factor (TF) of metals from the roots to shoots were calculated following Eqs. 1 and 2, respectively:

1 $$BAF_{Root} = {\raise0.7ex\hbox{${M_{Root} }$} \!\mathord{\left/ {\vphantom {{M_{Root} } {M_{soil} }}}\right.\kern-0pt} \!\lower0.7ex\hbox{${M_{soil} }$}}and \, BAF_{shoot} = {\raise0.7ex\hbox{${M_{shoot} }$} \!\mathord{\left/ {\vphantom {{M_{shoot} } {M_{soil} }}}\right.\kern-0pt} \!\lower0.7ex\hbox{${M_{soil} }$}}$$

2 $$TF = {\raise0.7ex\hbox{${M_{shoot} }$} \!\mathord{\left/ {\vphantom {{M_{shoot} } {M_{root} }}}\right.\kern-0pt} \!\lower0.7ex\hbox{${M_{root} }$}}$$

where, M Root is the metal concentration in the root, M Soil is the metal concentration in the soil and M shoot is the metal concentration in the aerial parts.

All metal concentrations in plant tissues are reported on a dry weight basis. When regulatory thresholds are defined on a fresh weight basis, concentrations were converted using the measured moisture content of the samples to allow for direct and accurate comparison with guideline values.

### Germination and morphometric parameters

Seedling emergence and early growth were evaluated in accordance with OECD Guideline 208 (2006). For each test soil, ten lettuce (*Lactuca sativa* L.) seeds were uniformly distributed on the soil surface in each pot and maintained under controlled conditions to support germination and growth. Distilled water was added daily to maintain 50% of the soil water-holding capacity. Seedling emergence was recorded after 7 days, and the germination rate (%) was calculated as the number of emerged seedlings divided by the total number of seeds sown, multiplied by 100 (Eq. 3).

The germination index (GI) was determined according to Zucconi et al. (1981) using the following equation:

3 $$GI = \frac{{seed ger\min ation_{ from each soil test} \times root length_{from each soil test} }}{{seed ger\min ation_{ from reference soil} \times root length_{ from reference soil} }} x100$$

After the exposure period, plants were carefully rinsed with distilled water to remove adhering soil particles. Endpoints included fresh biomass of roots and shoots (g) and shoot length, measured from the stem base to the apical meristem. The test duration was extended to 30 days to allow assessment of early growth and potential phytotoxic effects relative to the control.

Leaf necrosis, chlorosis, and root blackening were assessed using a semi-quantitative visual scoring system. For each treatment, the number of plants exhibiting visible symptoms was recorded, and results were expressed as the percentage of affected plants relative to the total number of individuals per experimental unit.

### Chlorophyll a, chlorophyll b and carotenoid contents

The chlorophyll *a*, chlorophyll *b*, and carotenoid contents were determined according to the Lichtenthaler and Welburn method (1983). In this method, 0.2 g of fresh plant tissue was pulverized with cryogenic liquid nitrogen, then extracted with acetone (80%) and filtered. The absorbance of the filtrate was measured spectrophotometrically at wavelengths of 646 and 663 nm. The concentrations of chlorophyll and carotenoid were calculated using the Eqs. 4.

$$chl_{a} = 12.25 \times A_{663} - 2.79 \times A_{646}$$

$$chl_{b} = 21.5 \times A_{646} - 5.10 \times A_{663}$$

4 $$Car = \left( {1000 \times A_{470} - 1.82 \times chl_{a} - 85.02 \times chl_{b} } \right)/198$$

where, Chl_a_ is the Chlorophyll *a*, Chl_b_ is the Chlorophyll *b*, Car is the carotenoid content and A_i_ is the absorbance of determinate wavelengths.

### Pattern and frequency of stomata on leaf surface

Stomatal traits were assessed 30 days after planting using fully expanded four-week-old leaves collected from both adaxial and abaxial epidermal surfaces. Stomatal frequency was quantified as the number of stomata per mm^2^ of epidermal area.

Stomatal morphology was classified into predefined quantitative categories based on guard cell size and structural integrity (compared to those found in the reference sites—PESRM and PES). Stomata were classified as normal when their size and shape fell within ± 10% of the mean guard cell dimensions observed in the reference areas and exhibited intact, symmetrically shaped guard cells. Big and small stomata were defined as individuals exhibiting guard cell dimensions greater than or smaller than this threshold, respectively. Defective stomata were identified by visible structural abnormalities, including malformed guard cells, incomplete pore formation, or irregular cell wall development. Open stomata were defined as those displaying a visibly open pore aperture at the time of observation.

For each treatment, four biological replicates were sampled at standardized leaf positions to minimize positional variability. Epidermal impressions were obtained using the transparent nail polish method (Zeng et al., 2008), and slides were examined under an optical microscope (Olympus DP71).

The relative frequency (%) of each stomatal category and total stomatal density were calculated per mm^2^, allowing quantitative comparisons among treatments and between epidermal surfaces.

### Statistical analysis

Differences between years were analyzed using an independent-samples t-test (two-tailed, p < 0.05), as comparisons involved two groups. Differences among study areas were evaluated using one-way analysis of variance (ANOVA) followed by Duncan’s multiple range post hoc test (p < 0.05) for comparisons among more than two groups.

Prior to parametric analyses, data were tested for normality using the Shapiro–Wilk test and for homogeneity of variances using Levene’s test. When these assumptions were not met, data were log-transformed to meet test requirements.

Statistical analyses were performed using the mean values of four replicates per treatment (n = 4) and conducted in SPSS version 18.0. Results are expressed as mean ± standard deviation (SD). Pearson’s correlation coefficient was used to assess relationships between soil metal bioavailability and metal bioaccumulation in roots and shoots.

## Results

### Metal bioavailability in soils

**Fig. 2:**
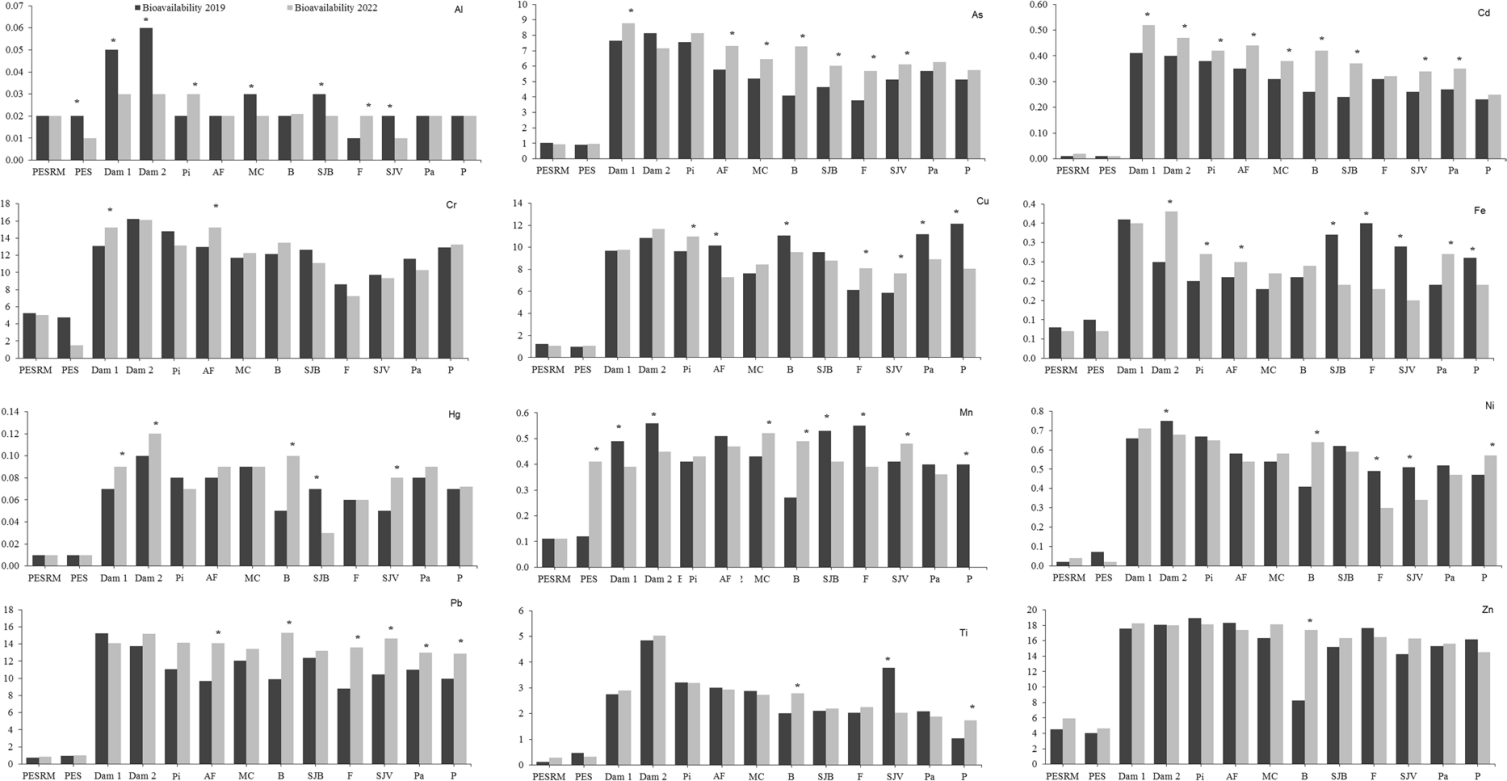
Metal bioavailability in soils affected by the Córrego do Feijão Mine dam rupture. Al and Fe are expressed as %, while all other metals are reported in mg kg⁻^1^ (dry weight). Acronyms refer to: PESRM-Parque Estadual da Serra do Rola Moça; PES-Parque Estadual do Sumidouro; Dam 1 and 2, dam areas; Pi-Pinheiros; AF-Alberto Flores; MC-Mário Campos; B-Betim; SJB-São Joaquim de Bicas; F-Florestal; SJV-São José da Varginha; Pa-Paraopeba; and P-Pompeu. Between sampling years, asterisks indicate statistically significant differences at p < 0.05 (n = 4)

Between the years 2019 and 2022, a small variability was noted in the bioavailable metal concentrations. The highest metal bioavailability in the affected areas was observed for Ti, Mn, Fe, Al, Zn, Pb, Cr, Cu, As, Ni, Cd, and Hg for both sampling years, as shown in Fig. 2. Increased bioavailability was related to higher metal contents in areas near the CFM dam, which were directly impacted by the tailing spill (Table S1).

### Concentration, bioaccumulation and translocation of metals in plants

**Fig. 3:**
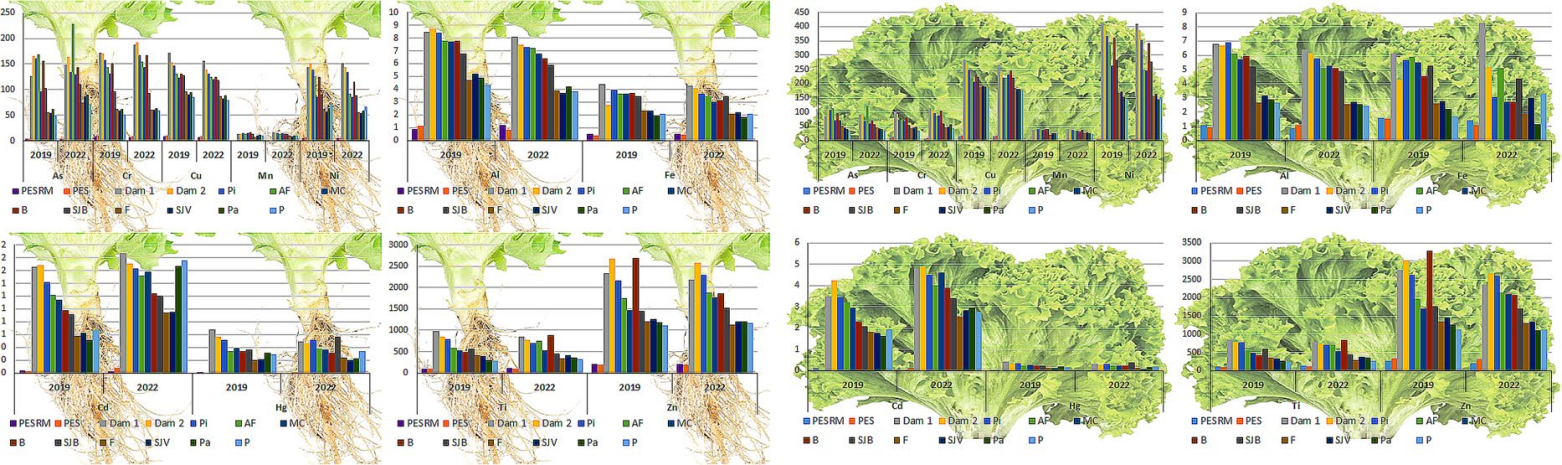
Total metal concentrations in the roots (A) and shoots (B) of lettuce (*Lactuca sativa* L.) cultivated in soils affected by the Córrego do Feijão Mine dam failure. Al and Fe are expressed as %, while all other metals are reported in mg kg⁻^1^ (dry weight). Acronyms refer to: PESRM-Parque Estadual da Serra do Rola Moça; PES-Parque Estadual do Sumidouro; Dam 1 and 2, dam areas; Pi-Pinheiros; AF-Alberto Flores; MC-Mário Campos; B-Betim; SJB-São Joaquim de Bicas; F-Florestal; SJV-São José da Varginha; Pa-Paraopeba; and P-Pompeu

Overall, metal concentrations in the lettuce tissues followed the highest levels in soils: Fe > Al > Mn > Ti > Cr > Cu > Pb > Zn > Ni > As > Cd > Hg (Fig. 3). In plants, Al, As, Cr, Hg, and Ti contents were higher in roots than in aerial parts (Fig. 3A). However, the highest contents of Cd, Cu, Fe, Mn, Ni, Pb, and Zn were observed in lettuce aerial parts (Fig. 3B). Low variability in the concentrations between the years was noted, especially for Cd, As, Mn, and Pb, suggesting that there was no improvement in soil conditions over three years. In shoots, the concentrations of As, Cd, Pb, and Cu, expressed on a fresh weight basis, were up to 407-, 98-, 5,483-, and 28-fold higher, respectively, than the maximum tolerated limits established by ANVISA (2021) for lettuce (Asteraceae) and other leafy vegetables. These levels also exceeded, by approximately two- to threefold, the maximum limits for leafy vegetables set by the European Union (EU, 2023) for Cd and As, and the guideline values established by FAO/WHO (2016) for As, Cd, Cu, and Pb.

**Fig. 4:**
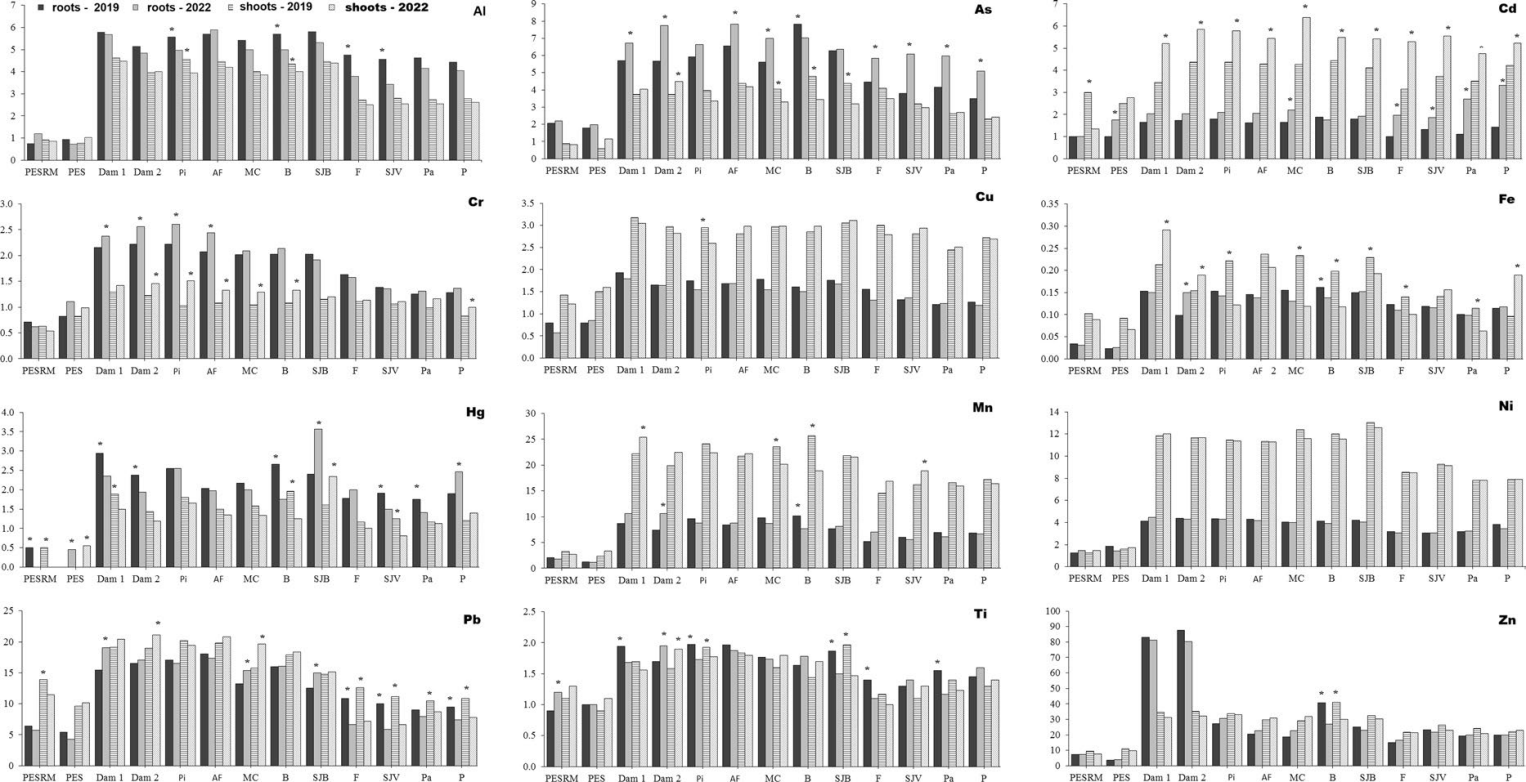
Metal bioaccumulation in the roots and shoots of lettuce (*Lactuca sativa* L.) cultivated in soils affected by the Córrego do Feijão Mine dam failure. Between sampling years, asterisks indicate statistically significant differences at p < 0.05 (n = 4). Acronyms refer to: PESRM-Parque Estadual da Serra do Rola Moça; PES-Parque Estadual do Sumidouro; Dam 1 and 2, dam areas; Pi-Pinheiros; AF-Alberto Flores; MC-Mário Campos; B-Betim; SJB-São Joaquim de Bicas; F-Florestal; SJV-São José da Varginha; Pa-Paraopeba; and P-Pompeu

The most bioaccumulated elements in lettuce grown in soils contaminated with CFM tailings followed the order: Zn > Pb > Cd > As > Mn > Hg > Al > Ni > Cu > Cr > Fe > Ti (Fig. 4), indicating a relationship with increasing metal concentrations in areas closer to the dam failure site. For Al, As, Cr, Hg and Ti, higher bioaccumulation factors BAFs were observed in roots, whereas for Cd, Cu, Fe, Mn, Pb, Ni, and Zn, the highest BAFs were recorded in the aerial parts (Fig. 4). Generally, BAF for Cd, As, Cr, Mn, Ni, Zn, and Pb were higher in 2022, in contrast to Al, Cu, Fe, Hg, and Ti, which exhibited higher BAFs in 2019. Among the elements most strongly bioaccumulated in tailings-affected areas, Zn, Mn, As, Cd, and Pb showed enrichment factors ranging from 15–86, 15–25, 3–8, 3–6, and 6–20 times, respectively.

**Fig. 5:**
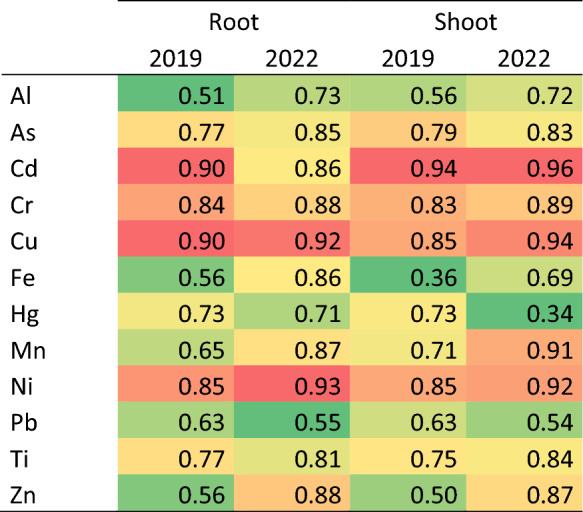
Heatmap of Spearman´s correlation coefficients between metal concentration in the mobile fraction of soil and metal accumulation in lettuce roots and shoots

Spearman’s correlation analysis revealed predominantly positive and statistically significant relationships between bioavailable metal concentrations in soils and their bioaccumulation in lettuce roots and shoots in both sampling years (Table S3; Fig. 5). In 2019, non-significant correlations were observed for Al in both roots and shoots (ρ = 0.280–0.402, p > 0.05), Fe in the aerial part (ρ = 0.322, p = 0.283), and Zn in roots (ρ = 0.407, p = 0.167), indicating a limited coupling between soil bioavailability and plant uptake for these elements. Conversely, Ni exhibited very strong correlations with bioaccumulation in both plant compartments (ρ = 0.897–0.907, p < 0.001), while As and Cd showed strong positive correlations in roots and shoots (ρ = 0.717–0.756, p < 0.05). Cr and Pb were strongly correlated with root bioaccumulation (ρ = 0.712–0.843, p < 0.01), whereas Cu displayed a stronger association with accumulation in the aerial tissues (ρ = 0.758, p = 0.003). The remaining elements generally presented moderate correlations.

In 2022, correlation strength increased for most elements and plant compartments, with only Zn in roots showing a non-significant relationship with soil bioavailability (ρ = 0.539, p = 0.057). Very strong correlations were recorded for As and Ni in both roots and shoots (ρ = 0.906–0.971, p < 0.001), while strong correlations were observed for Cd, Cr, Cu, Mn, and Ti (ρ = 0.741–0.865, p ≤ 0.01). Additionally, Fe, Hg, and Pb exhibited strong associations with root bioaccumulation (ρ = 0.700—0.807, p ≤ 0.02). The heatmap in Fig. 5 illustrates an overall strengthening of soil–plant coupling in 2022, particularly for potentially toxic elements, underscoring the relevance of the mobile soil fraction as a robust predictor of metal transfer to edible plant tissues. These findings highlight the importance of this relationship for environmental risk assessment and food safety.

**Fig. 6:**
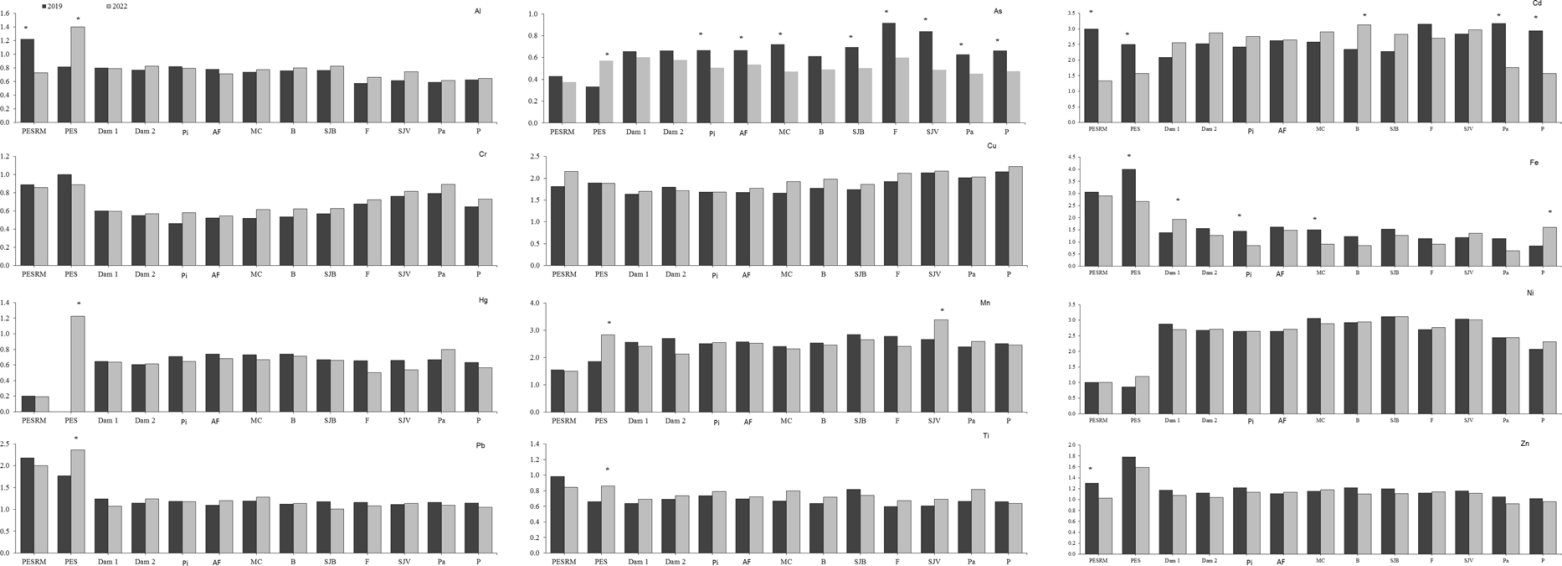
Metal translocation in lettuce (*Lactuca sativa* L.) cultivated in soils affected by the Córrego do Feijão Mine dam failure. Between sampling years, asterisks indicate statistically significant differences at p < 0.05 (n = 4). Acronyms refer to: PESRM-Parque Estadual da Serra do Rola Moça; PES-Parque Estadual do Sumidouro; Dam 1 and 2, dam areas; Pi-Pinheiros; AF-Alberto Flores; MC-Mário Campos; B-Betim; SJB-São Joaquim de Bicas; F-Florestal; SJV-São José da Varginha; Pa-Paraopeba; and P-Pompeu

Translocation factors of Ti, Al, As, Cr, and Hg were lower than 1, showing that the plant had low translocation ability for these trace elements, especially from the roots to the aerial parts. The highest translocation values were for Ni > Cd > Mn > Cu > Fe > Pb > Zn, indicating a higher concentration and bioaccumulation in the aerial parts than in the roots (TF > 1). A low variability in the metal translocation was observed between sampling years, except for As and Cd (Fig. 6).

### Effect of mine tailings on germination and morphometric features of lettuce plants

**Fig. 7:**
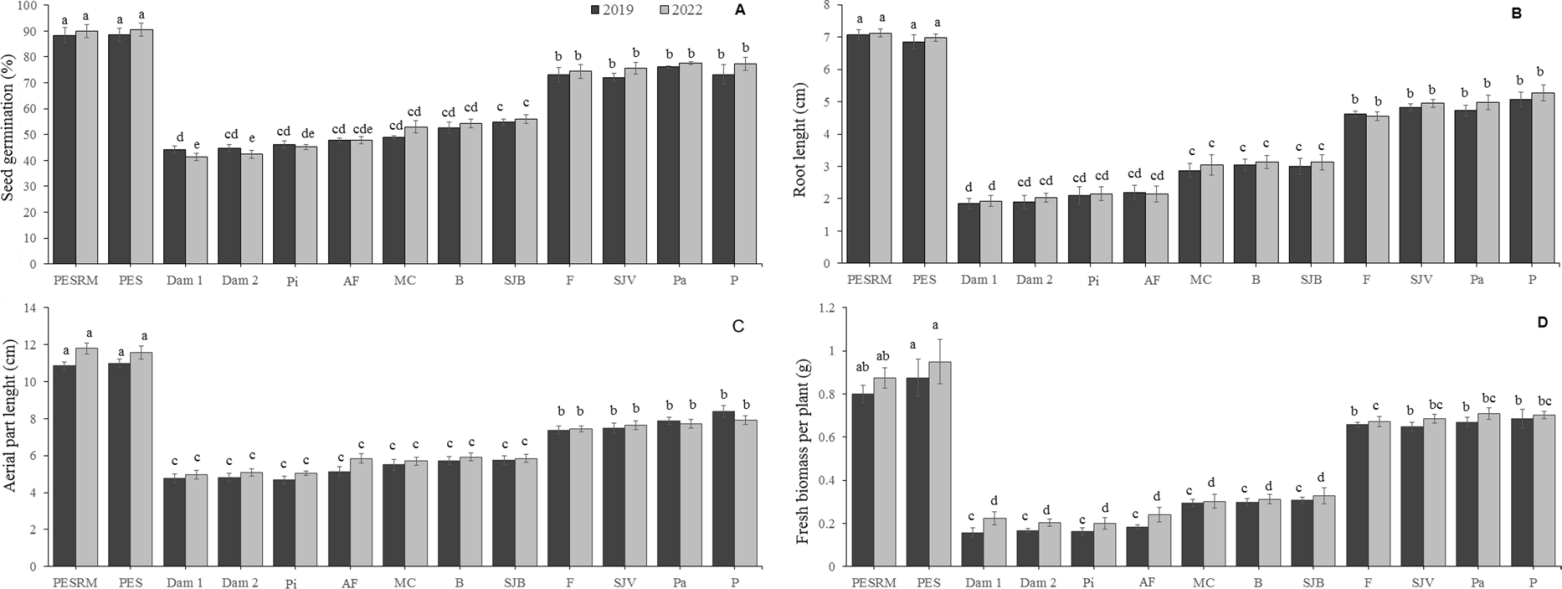
Metal effects in lettuce (*Lactuca sativa* L.). **A**-Seed germination. **B**-Root length. **C**-Aerial part length. **D**-Fresh biomass. Between areas, values with different letters are statistically different at p < 0.05 (n = 4). Acronyms refer to: PESRM-Parque Estadual da Serra do Rola Moça; PES-Parque Estadual do Sumidouro; Dam 1 and 2, dam areas; Pi-Pinheiros; AF-Alberto Flores; MC-Mário Campos; B-Betim; SJB-São Joaquim de Bicas; F-Florestal; SJV-São José da Varginha; Pa-Paraopeba; and P-Pompeu

Germination of *L. sativa* in the reference areas (PESRM and PES) was, on average, between 84 and 87% in 2019 and between 87 and 89% in 2022. In general, over two years of monitoring, lower germination rates (up to 52%) were observed in areas closest to the dam, such as Dam1, Dam 2, Pi, AF, MC, B, and SJB, which were directly affected by CFM tailings (Fig. 7A). In contrast, in areas partially affected by CFM tailings (F, SJV, Pa, and P areas), germination rates were higher than 70%.

Significant differences in the root length of lettuces were noted in the areas directly affected by CFM tailings, ranging from 1.7 cm (in Dam 1 area) to 3.4 cm (in SJB area), while the root length from reference areas presented an average of 7.0 cm and 6.7 cm (in PESRM and PES areas, respectively) (Fig. 7B). In the F, SJV, Pa, and P areas, the root length did not exceed 4.7 cm (average length). Regarding the aerial part length of *L. sativa*, there was the same trend found in roots, revealing the shortest lengths in the areas closest to those of CFM dam areas. On average, the aerial part length ranged from 4.5 cm (in Dam 1 area) to 8.1 cm (in P area), as shown in Fig. 7C. In both reference areas, aerial parts were observed up to 12.2 cm (average length). Fresh weights of *L. sativa* in areas closest to those of dam rupture, such as Dam 1 and 2, Pi, AF, MC, B, and SJB areas indicated the shortest average biomass from 0.16 g to 0.31 g (Fig. 7D).

Relating to the morphological traits of lettuce plants, leaf necrosis, chlorosis and blackening of root system were observed in up to 9%, 15%, 18%, respectively, from the Dam1, Dam2, Pi, and AF areas on the eighteenth day of the experimental assay.

**Fig. 8:**
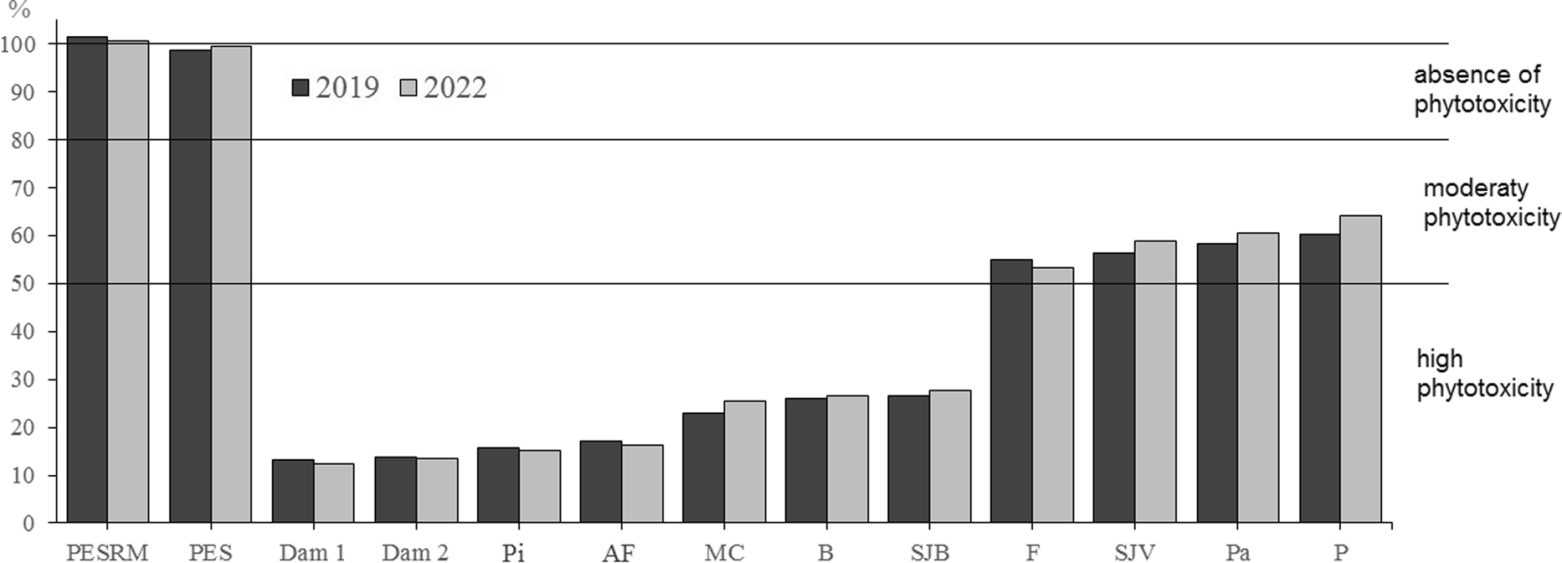
Germination index of lettuce (*Lactuca sativa* L.) cultivated in soils affected by the Córrego do Feijão Mine dam failure. Acronyms refer to: PESRM-Parque Estadual da Serra do Rola Moça; PES-Parque Estadual do Sumidouro; Dam 1 and 2, dam areas; Pi-Pinheiros; AF-Alberto Flores; MC-Mário Campos; B-Betim; SJB-São Joaquim de Bicas; F-Florestal; SJV-São José da Varginha; Pa-Paraopeba; and P-Pompeu

Based on the germination index (GI), the phytotoxicity in the studied areas can be classified into three levels: high phytotoxicity with GI < 50%, moderate phytotoxicity with 50% < GI < 80%, and absence of phytotoxicity with GI > 80% (Zucconi et al., 1981). Thus, areas closest to the dam failure, such as Dam1 and SJB areas, presented GIs of 13.2% and 26.7%, respectively, indicating high phytotoxicity levels (Fig. 8). In partially affected areas like F, SJV, Pa, and P, there was moderate phytotoxicity with GI values of 53% to 60%. In general, the tailings of the CFM dam in soils caused a significant reduction in GI.

### Chlorophyll a, chlorophyll b and carotenoid contents

**Fig. 9:**
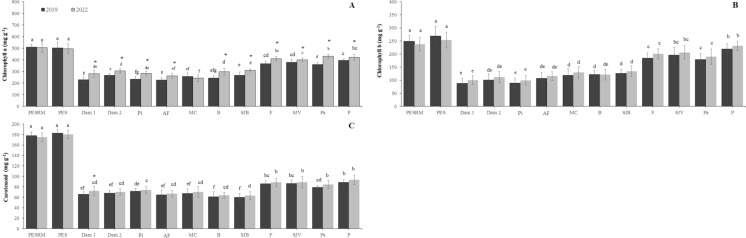
Pigment contents (fresh weight basis) in lettuce plants cultivated in soils affected by the Córrego do Feijão mine dam failure. (A) Chlorophyll *a*, (B) chlorophyll *b*, and (C) carotenoids. Different letters indicate statistically significant differences among areas, and asterisks indicate significant differences between sampling years (p < 0.05, n = 4).Acronyms refer to: PESRM-Parque Estadual da Serra do Rola Moça; PES-Parque Estadual do Sumidouro; Dam 1 and 2, dam areas; Pi-Pinheiros; AF-Alberto Flores; MC-Mário Campos; B-Betim; SJB-São Joaquim de Bicas; F-Florestal; SJV-São José da Varginha; Pa-Paraopeba; and P-Pompeu

The chlorophyll *a* (primary photosynthetic pigment) was significantly higher in the areas farther from the collapsed dam (F, SJV, Pa, and P areas), ranging from 382 mg g ^−1^ to 488 mg g ^−1^, while in Dam 1, Dam 2, Pi, AF, MC, B, and SJB areas had concentrations ranging from 202 mg g^−1^ to 307 mg g ^−1^. The pigmentation of chlorophyll *a* ranged from 508 mg g^−1^ to 518 mg g ^−1^ in the reference areas (Fig. 9A). Similarly, the chlorophyll *b* accessory pigmentation showed the lowest average values in areas close to the ruptured dam, ranging from 82 mg g^−1^ (in Pi area) to 152 mg g^−1^ (in SJB area). In contrast to what was observed in the reference areas with average values of 246 mg g^−1^ and 300 mg g^−1^ for PESRM and PES, respectively (Fig. 9B). Regarding carotenoids, all treatments showed significantly lower values than the reference areas, with concentrations of 178 mg g^−1^ for PESRM and 183 mg g^−1^ for PES (Fig. 9C). The areas directly affected by the CFM tailings showed the lowest values of carotenoids, as observed in the SJB area with average concentrations of 57 mg g^−1^. Overall, there was variability in the leaf pigmentation contents between the studied areas, showing less interference as they moved away from the collapsed dam. Furthermore, there was no difference between the years of sampling (Fig. 9).

### Pattern and frequency of stomata on leaf surface

In the reference areas, stomata on the adaxial and abaxial faces of lettuce leaves showed a number of 196 ± 5.0 and 241 ± 4.0 mm^2 −1^, respectively for PESRM area, and of 199 ± 6.1 and 232 ± 5.5 mm^2 −1^ for PES area, whose values were significantly higher than in directly affected areas (from 87 ± 3.9 to 133 ± 3.9 mm^2 −1^) and partially affected areas (from 137 ± 3.3 to 144 ± 4.4 mm^2 −1^) by CFM tailings (Table 2). The loss of normality pattern was greater in the areas closest to the dam, whose areas were totally affected by CFM tailings (Table 2). This proximity to the collapsed dam areas also influenced the number of open stomata, as seen in Dam 1 area, showing 58% and 50% in the adaxial and abaxial portions, respectively. Contrary to what has been observed in the reference areas with stomatal opening > 80%.

**Table 2:** Frequency of stomata (number per mm^2^) and their characteristics in lettuce leaves (adaxial and abaxial surface) developed in soils affected by tailings from the Córrego do Feijão Mine (Brumadinho, Brazil)

		Frequency	Normal (%)	Big (%)	Small (%)	Defective (%)	Open (%)
Adaxial	PESRM	196 (± 5.0) ^a^	94.4 (± 1.8) ^a^	1.1 (± 0.1) ^e^	1.5 (± 0.5) ^b^	3.1 (± 1.4) ^c^	84.5 (± 1.3) ^a^
	PES	199 (± 6.1) ^a^	94.5 (± 1.5) ^a^	1.5 (± 0.4) ^e^	1.5 (± 0.7) ^b^	2.5 (± 1.7) ^c^	82.8 (± 1.7) ^ab^
	Dam 1	87 (± 3.3) ^c^	63.9 (± 2.4) ^f^	8.1 (± 0.4) ^cd^	16.2 (± 2.4) ^a^	11.9 (± 3.9) ^a^	58.5 (± 4.7) ^d^
	Dam 2	89 (± 3.9) ^c^	65.7 (± 1.9) ^ef^	7.6 (± 0.2) ^cd^	15.8 (± 2.4) ^a^	10.9 (± 2.4) ^a^	59.3 (± 2.9) ^d^
	Pi	93 (± 3.7) ^c^	66.0 (± 2.1) ^def^	7.3 (± 0.3) ^cd^	15.5 (± 2.3) ^a^	11.2 (± 4.7) ^a^	61.5 (± 3.1) ^d^
	AF	94 (± 2.8) ^c^	70.1 (± 1.0) ^def^	7.2 (± 0.3) ^d^	13.4 (± 2.0) ^a^	9.3 (± 2.3) ^ab^	62.0 (± 2.4) ^d^
	MC	98 (± 3.5) ^c^	71.0 (± 1.3) ^de^	7.2 (± 0.3) ^d^	12.5 (± 2.4) ^a^	9.5 (± 1.6) ^a^	63.5 (± 2.4) ^d^
	B	101 (± 2.6) ^c^	69.8 (± 2.0) ^d^	6.7 (± 0.5) ^d^	12.9 (± 2.7) ^a^	10.6 (± 1.1) ^a^	64.8 (± 2.2) ^d^
	SJB	133 (± 3.9) ^b^	80.6 (± 1.9) ^c^	13.0 (± 2.3) ^a^	2.8 (± 0.9) ^b^	3.6 (± 0.7) ^bc^	74.3 (± 2.2) ^c^
	F	137 (± 3.3) ^b^	80.7 (± 1.1) ^bc^	13.1 (± 1.4) ^a^	2.9 (± 0.5) ^b^	3.4 (± 1.5) ^c^	74.5 (± 2.6) ^c^
	SJV	140 (± 3.4) ^b^	82.5 (± 1.9) ^bc^	11.7 (± 0.9) ^ab^	2.6 (± 1.5) ^b^	3.2 (± 2.0) ^c^	76.5 (± 2.4) ^bc^
	Pa	138 (± 3.7) ^b^	82.9 (± 2.0) ^bc^	11.2 (± 1.0) ^ab^	2.7 (± 2.0) ^b^	3.2 (± 2.6) ^c^	78.0 (± 2.4) ^abc^
	P	144 (± 4.4) ^b^	84.4 (± 1.4) ^b^	9.5 (± 0.5) ^bc^	2.7 (± 1.0) ^b^	3.3 (± 1.4) ^c^	78.1 (± 4.5) ^abc^
Abaxial	PESRM	241 (± 4.0) ^a^	90.3 (± 2.2) ^a^	2.6 (± 0.36) ^g^	3.3 (± 0.4) ^de^	3.8 (± 1.9) ^b^	81.8 (± 2.5) ^a^
	PES	232 (± 5.5) ^a^	89.3 (± 2.9) ^a^	2.8 (± 0.26) ^g^	4.8 (± 0.4) ^d^	3.2 (± 2.8) ^b^	79.5 (± 2.5) ^a^
	Dam 1	114 (± 4.4) ^c^	58.3 (± 2.9) ^c^	13.1 (± 0.57) ^de^	15.0 (± 0.6) ^a^	13.8 (± 3.8) ^a^	50.0 (± 2.6) ^d^
	Dam 2	117 (± 4.2) ^c^	59.7 (± 3.7) ^c^	12.4 (± 0.49) ^ef^	12.4 (± 0.8) ^bc^	16.9 (± 4.1) ^a^	49.5 (± 2.6) ^d^
	Pi	118 (± 5.3) ^c^	61.3 (± 2.8) ^c^	12.0 (± 0.68) ^ef^	12.2 (± 1.7) ^bc^	15.7 (± 3.6) ^a^	52.0 (± 2.6) ^d^
	AF	119 (± 4.5) ^c^	61.0 (± 3.7) ^c^	11.3 (± 0.79) ^ef^	12.5 (± 0.7) ^b^	14.5 (± 3.2) ^a^	52 (± 3.2) ^d^
	MC	121 (± 3.3) ^c^	63.9 (± 2.9) ^c^	11.0 (± 0.93) ^ef^	11.1 (± 0.5) ^bc^	14.0 (± 2.7) ^a^	53.2 (± 2.1) ^d^
	B	121 (± 4.7) ^c^	63.3 (± 2.9) ^c^	10.8 (± 0.83) ^f^	10.7 (± 0.3) ^c^	15.2 (± 3.1) ^a^	54.3 (± 2.2) ^d^
	SJB	147 (± 6.9) ^b^	75.6 (± 3.5) ^b^	19.3 (± 1.66) ^a^	2.4 (± 0.4) ^e^	2.8 (± 1.9) ^b^	64.3 (± 3.0) ^c^
	F	150 (± 5.3) ^b^	73.7 (± 3.1) ^b^	18.0 (± 0.79) ^ab^	3.4 (± 0.7) ^de^	4.9 (± 2.1) ^b^	65.5 (± 3.1) ^c^
	SJV	150 (± 4.7) ^b^	74.2 (± 2.5) ^b^	17.4 (± 0.78) ^ab^	3.8 (± 0.3) ^de^	4.7 (± 1.9) ^b^	67 (± 2.2) ^bc^
	Pa	152 (± 4.9) ^b^	74.4 (± 2.7) ^b^	16.5 (± 0.91) ^bc^	3.7 (± 0.4) ^de^	5.5 (± 3.4) ^b^	69.3 (± 1.7) ^bc^
	P	154 (± 4.0) ^b^	75.7 (± 2.1) ^b^	15.0 (± 0.87) ^cd^	3.4 (± 0.6) ^de^	6.5 (± 3.3) ^b^	72.7 (± 2.1) ^b^

Acronyms refer to: PESRM-Parque Estadual da Serra do Rola-Moça; PES-Parque Estadual do Sumidouro; Dam 1 and 2 to dam areas; Pi-Pinheiros; AF-Alberto Flores; MC-Mário Campos; B-Betim; SJB-São Joaquim de Bicas; F-Florestal; SJV-São José da Varginha; Pa-Paraopeba; P-Pompeu. Between areas, values with different letters are statistically different at p < 0.05 (n = 4)

## Discussion

Soils act as natural buffers regulating the dispersal and bioavailability of contaminants across atmospheric, aquatic, and biological compartments. While this buffering capacity may mitigate risks in non-agricultural settings, it becomes a critical concern in mining-impacted regions such as the State of Minas Gerais (Brazil), where agriculture and livestock production sustain local economies and where potentially toxic metals can accumulate and remain mobile within the soil matrix. Riparian soils are particularly influential due to their distinctive complexation and sorption dynamics (Buch et al., 2024a, 2024b). As aquatic-terrestrial ecotones, they exhibit unique biological, geomorphic, and biophysical attributes and provide essential ecosystem services, including chemical filtration, nutrient cycling, erosion control, flood mitigation, water purification, and regulation of hydrological flows (Buch et al., 2023).

In the present study, concentrations of all analyzed metals in riparian soils exceeded threshold values established by Brazilian regulations (CONAMA, 2009; COPAM-MG, 2011) and international environmental guidelines (Zarcinas et al., 2004; CCME, 2007; MEF, 2007; Tóth et al., 2016; Buch et al., 2023). Elevated levels of As (> 8 mg kg⁻^1^), Cd (> 0.4 mg kg⁻^1^), Cr (> 80 mg kg⁻^1^), Cu (> 90 mg kg⁻^1^), Hg (> 0.2 mg kg⁻^1^), Ni (> 34 mg kg⁻^1^), and Pb (> 80 mg kg⁻^1^) observed in riparian soil samples were comparable to those reported for surface waters and sediments from the same region (CPRM, 2019a, 2019b; Pacheco et al., 2022; Teramoto et al., 2021; Thompson et al., 2020), indicating strong connectivity and metal transfer among environmental compartments. Under similar contamination scenarios, soils located farther from riverbanks typically display lower metal concentrations, with progressive declines observed with increasing depth (Liu et al., 2019; Ye et al., 2013). For example, riparian soils in southern Canada exhibited Pb concentrations up to twelve times higher than nearby agricultural soils (Saint-Laurent et al., 2010). Comparable spatial patterns have been reported in areas impacted by Córrego do Feijão (CFM) tailings, where hydrological drivers such as rainfall, surface runoff, water level fluctuations, and flow regulation control the redistribution and persistence of metals in soil profiles.

In riparian systems, both total metal concentrations and their chemical speciation strongly influence bioavailability (Kabata-Pendias, 2011; Zhong et al., 2022). Under acidic conditions, as observed in this study, As, Cd, Ni, and Zn tend to occur in higher proportions as free ionic species in the soil solution, enhancing their mobility and bioavailability (Kanianska et al., 2022; Pan et al., 2018). In contrast, Cr and Pb are more frequently associated with organic and inorganic ligands, forming relatively stable complexes that limit their mobility (Kim et al., 2015; Xia et al., 2018). These processes are governed by site-specific soil properties, including pH, redox potential, cation exchange capacity, clay mineralogy, and organic matter content (Buch et al., 2021). In Brazilian soils, kaolinite, Fe and Al oxides, and the humic fraction dominate the colloidal phase (Bettiol et al., 2023) and play a central role in metal immobilization through specific adsorption mechanisms characterized by strong covalent bonding to hydroxylated mineral surfaces (Palansooriya et al., 2020; Srivastava et al., 2005). In contrast, nonspecific electrostatic interactions typical of secondary 2:1 phyllosilicates exhibit lower binding energies, facilitating metal exchange and increasing the potential for mobilization and groundwater contamination (Buch et al., 2021; Zhang et al., 2014).

Plant roots can be regarded as selective sinks for ions in the soil solution. Metals may enter roots through multiple pathways (Peijnenburg et al., 2000). The predominant mechanism is uptake from the soil solution, preceded by transport to the root surface via mass flow associated with transpiration-driven water uptake or by diffusion through the soil solution along concentration gradients generated by the selective absorption of metal ions by roots (Barber, 1995). Consequently, the metal flux to the root is a function of both the volume of water absorbed and the metal concentration in the soil solution. When the rate of metal uptake by roots exceeds the supply delivered by mass flow, depletion zones can develop in the rhizosphere, promoting the diffusion of metals toward the root surface in response to the resulting concentration gradients (Peijnenburg et al., 2000).

On average, arsenic (As) accumulation in *L. sativa* roots is high, ranging from 77 to 92% across soil As concentrations between 40 mg g^−1^ and 150 mg g^−1^ (Smith et al., 2009). Consistent with the present study, several authors have reported significantly higher As concentrations in roots than in shoots, with root levels being 19–26 times greater, indicating a limited capacity for metal translocation to aerial tissues (Chang et al., 2014; Kumwimba et al., 2013; Yañez et al., 2019). This restricted mobility is commonly attributed to the intracellular complexation of As with phytochelatins and other thiol-rich ligands in root cells, which reduces efflux to the rhizosphere and limits xylem loading and subsequent transport to aerial parts (Liu et al., 2010). According to Huang et al. (2006), increasing As concentrations in soil are generally associated with a decrease in the translocation factor, as plants activate physiological exclusion and detoxification mechanisms to avoid toxicity. In contrast, methylated arsenic species tend to be less efficiently taken up by roots but are more readily translocated to shoots once absorbed, reflecting their higher mobility in the xylem sap-aerial parts (Raab et al., 2007; Zhang et al., 2024). Similar to As, aluminum (Al) and cadmium (Cd) in this study exhibited translocation factors < 1 and preferential accumulation in roots rather than in aerial tissues, suggesting low internal mobility and a strong root retention capacity in lettuce plants (Pérez-Figueroa et al., 2023). Comparable patterns have been reported for lettuces cultivated in soils impacted by the Fundão mine tailings dam collapse in Mariana, Brazil, where root tissues acted as primary sinks for potentially toxic elements (Bandeira et al., 2022). Aluminum, the third most abundant element in the Earth’s crust, is predominantly present in soils as insoluble minerals such as gibbsite and aluminosilicates, whose stability is strongly regulated by soil pH (Bojórquez-Quintal et al., 2017). Under acidic conditions (pH < 5), these forms can be solubilized into the phytotoxic Al^3^⁺ species in the soil solution, thereby enhancing its bioavailability and uptake by plant roots (Kochian et al., 2015). In mining-affected areas of Brazil, acidic soil conditions have been widely reported and are associated with increased Al solubilization and bioaccumulation, ultimately leading to phytotoxic effects and potential risks to food safety (Bandeira et al., 2022; Buch et al., 2021, 2023).

The differential relationships observed between soil bioavailability and plant bioaccumulation across elements may be partly explained by competitive interactions at the level of uptake transporters and nutrient acquisition pathways. Essential trace metals such as Fe, Zn, Mn, and Cu share common transport systems and regulatory mechanisms in plants, which can lead to competition both among essential elements and between essential and non-essential (potentially toxic) metals for uptake sites in the root system. Transporters such as members of the ZIP (ZRT/IRT-like Protein) family are known to facilitate the uptake of divalent cations including Zn, Fe, and Mn; these transporters are also reported to be non-specific, allowing entry of toxic metals such as Cd and Ni, which can compete with essential elements for binding and transport, thereby influencing accumulation patterns in plant tissues (Panda et al., 2025).

This competitive uptake mechanism is consistent with studies demonstrating that the presence of one metal can affect the absorption of another. For example, Cd has been shown to compete with Fe and Zn for uptake pathways, leading to altered acquisition and homeostasis of these micronutrients under metal stress conditions. Elevated concentrations of certain ions in the rhizosphere may suppress the uptake of others by saturating or inhibiting shared transporters, resulting in the preferential accumulation of metals with higher affinity for these transport systems. Moreover, interference can occur not only at the transporter level but also via modification of root development and rhizospheric chemistry, which can indirectly shape the uptake dynamics of multiple elements simultaneously (Lešková et al., 2022).

In the context of our results, the heightened correlations for Ni, As, and Cd in 2022 could reflect increased competitive advantage of these metals for shared uptake pathways under altered soil conditions, possibly due to shifts in soil chemistry affecting ligand availability and transporter expression. Conversely, weaker relationships observed for elements such as Fe and Zn in 2019 might be attributable to competition effects limiting their effective uptake despite their bioavailability in the soil. These findings emphasize the complexity of elemental interactions during uptake, where competitive binding, transporter specificity, and soil–root interface processes collectively determine the bioaccumulation patterns observed in edible plant tissues. (Umar et al., 2025).

Exposure to potentially toxic metals adversely affects plant growth and productivity from early developmental stages, including seed germination (Collins & Kinsela, 2011; Haider et al., 2021). Reported phytotoxic responses include morphological and physiological alterations in root systems, such as reduced primary and lateral root development, increased root dieback, and decreased root hair density and surface area. These effects have been documented for a wide range of elements, including Ag (Anjum et al., 2013; Noor et al., 2022), As (Garg & Singla, 2011; Panda et al., 2010), Cd (Loi et al., 2018), Co (Chatterjee & Chatterjee, 2000; Palit et al., 1994), Cr (Dias et al., 2016; Park, 2020), Cu (Wolf et al., 2017), Hg (Chen & Yang, 2012; León-Cañedo et al., 2019), Ni (Hawrylak-Nowak & Matraszek-Gawron, 2020), Pb (Silva et al., 2017; Ikkonen et al., 2022), and Zn (Broadley et al., 2007; Chaney, 1993; Wolf et al., 2017). Such metal-induced alterations compromise root–soil interactions, thereby limiting water and nutrient acquisition and ultimately constraining plant growth and yield (Okereafor et al., 2020).

Beyond root-level effects, metals disrupt nutrient uptake, alter plasma membrane integrity, and damage chloroplast ultrastructure, leading to changes in respiration and transpiration rates, enhanced generation of reactive oxygen species (ROS), and the activation of enzymatic and non-enzymatic antioxidant defenses. These processes impair photosynthetic performance, disturb water relations and mineral nutrition, and modulate hormonal homeostasis, resulting in widespread physiological dysfunction (Umar et al., 2025).

Low leaf pigment levels are commonly associated with environmental stressors such as drought and pollution (Agathokleous et al., 2019, 2020). Under metal stress, metabolic resources are diverted toward detoxification and sequestration, interfering with enzymatic pathways, accelerating pigment degradation, and suppressing de novo pigment biosynthesis.

Chlorophyll degradation is a regulated catabolic process in higher plants, typically activated during senescence to detoxify phototoxic pigments and recycle nutrients; however, chlorophyll catabolites may also serve physiological and ecological functions (Hörtensteiner & Kräutler, 2011). Chlorophyllase, which hydrolyzes chlorophyll to chlorophyllide, has been implicated in plant defense, including the suppression of chewing herbivores (Hu et al., 2015). Accordingly, reduced chlorophyllase activity is often associated with lower leaf pigment concentrations. In metal-stressed plants, declines commonly occur in the order chlorophyll *a* > chlorophyll *b* > carotenoids, as reported for faba bean (*Vicia faba* cv. Aštar), cucumber (*Cucumis sativus* L.), and cherry tomato (*Solanum lycopersicum* L.) exposed to elevated Cu, Pb, and Cd levels, respectively (Burzyński & Kłobus, 2004; Piršelová et al., 2016; Xie et al., 2021).

The phenomenon of hormesis, characterized by biphasic dose–response relationships in which low stressor doses stimulate and high doses inhibit biological traits, has been widely reported in metal-contaminated systems (Mattson, 2008). Under high-dose exposure, chlorophyll content generally declines alongside other physiological parameters (Agathokleous et al., 2020). Consistent with this pattern, a negative relationship between chlorophyll content, degradation, and chlorophyllase activity has been described (Siddiqui et al., 2019). These findings suggest that low-dose stimulation reflects the activation of anabolic pathways, whereas high-dose stress favors catabolic processes mediated by chlorophyllase (Calabrese et al., 2005). This framework highlights the need to determine whether low-dose-induced chlorophyll accumulation involves intermediate anabolic compounds that may also contribute to plant defense, for example by exerting deterrent or toxic effects on herbivores (Agathokleous et al., 2020; Hörtensteiner, 2018).

Metal interference in lettuce metabolism is further evidenced by reduced stomatal aperture, frequency, and size, particularly in plants cultivated in soils proximal to tailings dams. Metals may impair water transport to shoots by reducing leaf area and lamina thickness, decreasing intercellular spaces, altering stomatal density, and limiting stomatal opening (Costa & Morel, 1994; Neelu et al., 2000; Pérez-Figueroa et al., 2023). Reports of stomatal reduction associated with decreased guard cell size in lettuce remain limited, and most available studies focus on one or two metals rather than complex mixtures typical of mining tailings (Balal et al., 2024; Costa & Morel, 1994; Sagardoy et al., 2010). Stomatal closure may result from direct interactions between metals and guard cells or from secondary responses to metal-induced damage in roots and stems. In this context, root-derived or exogenously induced abscisic acid (ABA) signaling has been proposed as a central regulator of stomatal movement under metal stress (Poschenrieder et al., 1989; Rucińska-Sobkowiak, 2016). Defective stomata in vegetables exposed to heavy metals may remain partially or permanently closed, reflecting the disorganization of cytoplasmic organelles within epidermal cells (Barceló et al., 1988). Such structural and functional impairments disrupt the regulation of stomatal dynamics, thereby limiting phenotypic plasticity and adaptive responses to water stress imposed by metal toxicity (Gupta & Bhatnagar, 2015).

Furthermore, disturbances in plant water relations can differentially regulate aquaporin gene expression, potentially contributing to additional constraints on water fluxes and stomatal frequency. It is noteworthy that heavy metal ions rarely reach solution concentrations sufficient to induce purely osmotic stress in plants without first eliciting symptoms of acute or chronic toxicity (Rucińska-Sobkowiak, 2016). Conversely, increased stomatal density per unit leaf area has been reported in *Helianthus annuus* exposed to Pb, Cd, Cu, and Zn (Kastori et al., 1992) and in *Beta vulgaris* spp. exposed to Cd (Greger & Johansson, 1992). This apparent increase is attributed to reduced guard cell size caused by metal-induced leaf water deficits, rather than to enhanced stomatal differentiation (Neelu et al., 2000; Wainwright & Woolhouse, 1977).

In general lettuce tends to accumulate more metals than other vegetables (e.g., onion, carrot, potato, beetroot, cabbage, spring onion, and tomato) due to its large surface area, which enhances its ability to absorb atmospheric deposits and through its high transpiration rate, which draws metals from the soil (Aina et al., 2024; Alegbe et al., 2025; Hadayat et al., 2018; Wagner, 1993). This, coupled with its classification as a hyperaccumulator, means lettuce can tolerate and concentrate metals in its tissues, particularly in the leaves (Alegbe et al., 2025; Dala-Paula et al., 2018). Bioaccumulation of heavy metals in vegetables poses an enormous health risk to animals and humans, being prominently reported in recent years (Oyewumi et al., 2025; Yang et al., 2025). The As, Cr, Cd and Pb are classified as human carcinogens, with children and women being the most susceptible groups (Hadayat et al., 2018; USEPA, 2005). These metals can accumulate in tissues for a long time due to its affinity with brain, kidney and muscle cells, what can cause multiple damages to human health, such as cause kidney, bone and respiratory diseases and liver damage (Balali-Mood et al., 2021; Sharafi et al., 2019). As previously mentioned, the levels of metals found in shoots were mostly above the tolerable limits for human health (FAO/WHO, 2016; ANVISA, 2021; EU, 2023). In previous studies conducted in the same areas, soils affected by mine tailings from the Córrego do Feijão (CFM) and Fundão dam failures in Minas Gerais, Brazil, exhibited a high potential for both carcinogenic and non-carcinogenic health risks (Buch et al., 2024a, 2024b). Based on hazard indices derived from chronic daily intake across multiple exposure pathways, including incidental soil ingestion and crop consumption, As, Cd, Cr, Ni, and Pb were identified as the primary contributors to cancer risk in both children and adults (Buch et al., 2024a, 2024b). Together with the findings of the present study, these results underscore the need for intervention measures to restore and mitigate severely affected areas and to reduce adverse effects on human health. Furthermore, strengthened environmental policies and regulatory directives are essential to protect the entire trophic chain, particularly in mining-impacted regions.

## Conclusion

Vegetables represent a critical source of essential nutrients in the human diet. However, their nutritional value is intrinsically linked to the environmental quality of the soils in which they are cultivated. This study highlights that in regions influenced by geological enrichment and, more critically, by mining activities, soil–plant interactions can transform food crops into effective pathways for human exposure to potentially toxic elements. Few studies have evaluated lettuce growth in soils contaminated with mining waste, and those that do typically focus on the effects of only one to three metals. Our findings demonstrate that metal mobility and plant uptake are governed not only by total soil concentrations but also by soil physicochemical conditions and metal speciation, which regulate bioavailability and translocation within plant tissues. Furthermore, metal translocation is influenced by interactions with abiotic and biotic components, particularly plant species, which differ in physiology, morphology, and other species-specific traits.

From a public health perspective, the substantial transfer of multiple metals from roots to edible tissues in lettuce underscores the vulnerability of raw-consumed leafy vegetables as vectors of dietary exposure, particularly in regions with ongoing or legacy mining activities. These results emphasize the importance of integrating geochemical assessment, plant physiology, and toxicological criteria into risk assessment frameworks, rather than relying solely on bulk soil or plant concentration metrics. Future research should expand to multi-season and multi-crop assessments, incorporate metal speciation in plant tissues, and evaluate the effectiveness of soil amendments and phytoremediation strategies in reducing metal bioavailability.

Regarding soils affected by mining tailings following the dam collapse, although the most pronounced adverse effects were observed in areas closest to the dams (Dam 1, Dam 2, Pi, AF, MC, and B), impairments in soil health and lettuce development were evident across all assessed sites. Therefore, these areas require remedial actions and the implementation of mitigation measures to ensure food safety and protect animal and human health.

## Supplementary Information

Below is the link to the electronic supplementary material.

Supplementary file1 (DOCX 17 KB)

Supplementary file2 (DOCX 17 KB)

Supplementary file3 (DOCX 15 KB)

## Data Availability

Data will be made available on request.
