# Correction: Phytotoxicity and metal mobility in soils contaminated with mine tailings

**DOI:** 10.1007/s10653-026-03245-1

**Published:** 2026-05-27

**Authors:** Andressa Cristhy Buch, Maria Elizabeth Fernandes Correia, Júlia Carina Niemeyer, Douglas Brian Sims, Eduardo Duarte Marques, Krishna Kumar Yadav, Ahmad J. Obaidullah, Camila Rodrigues e Silva, Emmanoel Vieira da Silva-Filho

**Affiliations:** 1 Department of Environmental Geochemistry, Universidade Federal Fluminense, Outeiro São João Baptista, S/N, Centro, Niterói, Rio de Janeiro 24020-007 Brazil; 2 Brazilian Agricultural Research Company (EMBRAPA), Seropédica, Rio de Janeiro 23897-000 Brazil; 3 Postgraduate Program in Agricultural and Natural Ecosystems, Universidade de Santa Catarina, Campus de Curitibanos, Rod. Ulysses Gabordi, km 3, Curitibanos, Santa Catarina 89520-000 Brazil; 4 Department of Physical Sciences, College of Southern Nevada, North Las Vegas, NV 89030 USA; 5 Service Geological Survey of Brazil/Company of Research of Mineral Resources (SGB/CPRM), Av. Brasil, 1731, Funcionários, Belo Horizonte, Minas Gerais 30140-002 Brazil; 6 Department of VLSI Microelectronics, Saveetha School of Engineering, Saveetha Institute of Medical and Technical Sciences (SIMATS), Saveetha University, Chennai, Tamil Nadu 602105 India; 7 Environmental and Atmospheric Sciences Research Group, Scientific Research Center, Al-Ayen University, Nasiriyah, Thi-Qar Iraq; 8 Department of Pharmaceutical Chemistry, College of Pharmacy, King Saud University, P.O. Box 2457, 11451 Riyadh, Saudi Arabia; 9 Department of Chemistry, Graphic Era University, Dehradun, Uttarakhand 248002 India

**Correction to: Environ Geochem Health (2026) 48:313** 10.1007/s10653-026-03203-x

In the original publication, the author Maria Elizabeth Fernandes Correia was inadvertently omitted from the author group.

The author Maria Elizabeth Fernandes Correia is affiliated with the Brazilian Agricultural Research Company (EMBRAPA), Seropédica, Rio de Janeiro 23897-000, Brazil.

The original article has been corrected.

