# Supplementary material for: Phytotoxicity and metal mobility in soils contaminated with mine tailings
Source: Environ Geochem Health. 2026 Apr 24;48(7):313. doi: 10.1007/s10653-026-03203-x (PMC13109235; doi:10.1007/s10653-026-03203-x)
Supplement: Supplementary file 1 — Supplementary file1 (DOCX 17 KB) [file 10653_2026_3203_MOESM1_ESM.docx]

Table S1. Soil sampling areas after the dam failure of the Córrego do Feijão Mine (Brumadinho, Brazil).

| Areas/Municipality | Acronym | Distance of the dam (km) |  | Geographic coordinates | | |
| --- | --- | --- | --- | --- | --- | --- |
|  |  |  | altitude | longitude | | latitude |
| *Dam 1/Brumadinho | Dam 1 | 0 | 894 | -44.1178 | -20.1186 | |
| *Dam 2/Brumadinho | Dam 2 | 1.5 | 850 | -44.1311 | -20.1412 | |
| *Pi-Pinheiros /Brumadinho | Brum 1 | 6 | 833 | -44.1622 | -20.1744 | |
| *AF-Alberto Flores/Brumadinho | Brum 2 | 10 | 827 | -44.1852 | -20.1445 | |
| *MC-Mário Campos | MC | 16 | 821 | -44.2097 | -20.1236 | |
| *B-Betim | B | 29 | 813 | -44.2732 | -19.9888 | |
| **SJB-São Joaquim de Bicas | SJB | 31 | 751 | -44.2041 | -20.0588 | |
| **F-Florestal | F | 57 | 749 | -44.3799 | -19.8606 | |
| **SJV-São José da Varginha | SJV | 95 | 746 | -44.4998 | -19.6606 | |
| **Pa-Paraopeba | Pa | 130 | 665 | -44.5376 | -19.3275 | |
| **P-Pompeu | P | 180 | 624 | -44.7803 | -19.0022 | |
| *** Parque Estadual da Serra do Rola-Moça/Nova Lima | PESRM | 24 | 828 | -44.0015 | -20.0487 | |
| *** Parque Estadual do Sumidouro/ Pedro Leopoldo and Lagoa Santa | PES | 130 | 742 | -43.9592 | -19.5622 | |

* The asterisk indicates the areas directly impacted by the mining mud from Córrego do Feijão; **partially affected areas correspond to areas that came into contact with ore tailings after the rivers overflowed due to the rains; *** unaffected areas: references.
