# Supplementary material for: Phytotoxicity and metal mobility in soils contaminated with mine tailings
Source: Environ Geochem Health. 2026 Apr 24;48(7):313. doi: 10.1007/s10653-026-03203-x (PMC13109235; doi:10.1007/s10653-026-03203-x)
Supplement: Supplementary file 2 — Supplementary file2 (DOCX 17 KB) [file 10653_2026_3203_MOESM2_ESM.docx]

Table S2. Physical-chemical characterization of soils affected by the dam failure of the Córrego do Feijão Mine (Brumadinho, Brazil).

| Areas | sand | | silt | | clay | | bulk density | | pH | | Organic matter | |
| --- | --- | --- | --- | --- | --- | --- | --- | --- | --- | --- | --- | --- |
|  | % | | % | | % | | g cm^-3^ | |  | | % | |
|  | 2019 | 2022 | 2019 | 2022 | 2019 | 2022 | 2019 | 2022 | 2019 | 2022 | 2019 | 2022 |
| Dam 1 | 64 | 66 | 18 | 18 | 18 | 16 | 4.27 | 4.85 | 2.9 | 3.6 | 1.35 | 1.41 |
| Dam 2 | 62 | 64 | 22 | 22 | 16 | 14 | 4.31 | 4.63 | 3.1 | 3.5 | 0.78 | 1.00 |
| Pi | 60 | 62 | 20 | 19 | 20 | 19 | 3.41 | 3.98 | 3.4 | 3.8 | 1.02 | 1.07 |
| AF | 57 | 55 | 18 | 22 | 25 | 23 | 3.27 | 4.12 | 4.5 | 4.0 | 1.69 | 1.74 |
| MC | 54 | 52 | 18 | 23 | 28 | 25 | 3.42 | 3.89 | 3.9 | 3.5 | 2.31 | 2.15 |
| B | 54 | 58 | 20 | 20 | 26 | 22 | 3.32 | 3.76 | 3.5 | 3.2 | 2.17 | 2.02 |
| SJB | 59 | 57 | 19 | 23 | 22 | 20 | 1.98 | 1.87 | 4.7 | 4.1 | 2.98 | 3.14 |
| Flo | 55 | 56 | 19 | 19 | 26 | 25 | 2.10 | 1.70 | 4.4 | 4.2 | 4.34 | 4.28 |
| SJV | 42 | 44 | 28 | 24 | 30 | 32 | 1.72 | 1.78 | 3.7 | 3.9 | 6.34 | 6.14 |
| Pa | 53 | 54 | 28 | 29 | 19 | 17 | 1.84 | 1.79 | 4.1 | 3.7 | 4.24 | 4.33 |
| P | 44 | 47 | 20 | 16 | 36 | 37 | 1.65 | 1.72 | 4.1 | 3.8 | 7.52 | 7.64 |
| PESRM | 38 | 39 | 20 | 17 | 42 | 44 | 1.31 | 1.35 | 4.8 | 4.9 | 9.99 | 10.02 |
| PES | 27 | 29 | 27 | 26 | 46 | 45 | 1.22 | 1.30 | 5.2 | 5.7 | 11.38 | 11.40 |

Dam 1 and 2 refers to dam areas; Pi-Pinheiros; AF-Alberto Flores; MC-Mário Campos; B-Betim; SJB-São Joaquim de Bicas; F-Florestal; SJV-São José da Varginha; Pa- Paraopeba; P-Pompeu; PESRM-Parque Estadual da Serra do Rola-Moça; PES-Parque Estadual do Sumidouro.
