# Supplementary material for: Phytotoxicity and metal mobility in soils contaminated with mine tailings
Source: Environ Geochem Health. 2026 Apr 24;48(7):313. doi: 10.1007/s10653-026-03203-x (PMC13109235; doi:10.1007/s10653-026-03203-x)
Supplement: Supplementary file 3 — Supplementary file3 (DOCX 15 KB) [file 10653_2026_3203_MOESM3_ESM.docx]

Table S3. Spearman’s correlation coefficients between metal bioavailability in soils and bioaccumulation in plant roots and shoots. Values between parentheses are p-values.

|  | 2019 | | 2022 | |
| --- | --- | --- | --- | --- |
|  | Relation in root | Relation in aerial part | Relation in root | Relation in aerial part |
| Al | 0.280 (0.353) | 0.402 (0.173) | 0.593 (0.033) | 0.613 (0.026) |
| As | 0.717 (0.025) | 0.739 (0.019) | 0.932 (0.000) | 0.906 (0.000) |
| Cd | 0.734 (0.020) | 0.756 (0.015) | 0.706 (0.010) | 0.865 (0.000) |
| Cr | 0.843 (0.000) | 0.680 (0.010) | 0.820 (0.001) | 0.741 (0.004) |
| Cu | 0.671 (0.012) | 0.758 (0.003) | 0.848 (0.000) | 0.842 (0.000) |
| Fe | 0.618 (0.024) | 0.322 (0.283) | 0.788 (0.001) | 0.561 (0.046) |
| Hg | 0.633 (0.027) | 0.543 (0.068) | 0.753 (0.007) | 0.626 (0.017) |
| Mn | 0.591 (0.033) | 0.676 (0.011) | 0.797 (0.001) | 0.864 (0.000) |
| Ni | 0.907 (0.000) | 0.897 (0.000) | 0.971 (0.000) | 0.924 (0.000) |
| Pb | 0.712 (0.006) | 0.485 (0.093) | 0.700 (0.020) | 0.537 (0.061) |
| Ti | 0.630 (0.021) | 0.498 (0.083) | 0.807 (0.001) | 0.752 (0.003) |
| Zn | 0.407 (0.167) | 0.571 (0.041) | 0.539 (0.057) | 0.928 (0.000) |
